# The Random Domino Automaton on the Bethe Lattice and Power-Law Cluster-Size Distributions

**DOI:** 10.3390/e27121226

**Published:** 2025-12-03

**Authors:** Mariusz Białecki, Arpan Bagchi, Yohei Tutiya

**Affiliations:** 1 Institute of Geophysics Polish Academy of Sciences, Ks. Janusza 64, 01-452 Warszawa, Poland; 2 Center for Basic Education and Integrated Learning, Kanagawa Institute of Technology, Shimo-Ogino 1030, Atsugi 243-0292, Kanagawa, Japan; tutiya@gen.kanagawa-it.ac.jp

**Keywords:** self-organized criticality, inverse-power distribution, stochastic dynamics, solvable model, forest-fire models, Bethe-lattice, Catalan-like recurrences, cellular automata, stationary Markov process

## Abstract

The Random Domino Automaton—a stochastic cellular automaton forest-fire model—is formulated for the Bethe lattice geometry. The equations describing the stationary state of the system are derived using combinatorial analysis. The special choice of parameters that define the dynamics of the system leads to a solvable reduction in the set of equations. Analysis of the equations shows that by changing the parameter responsible for cluster removal, the size distribution of clusters smoothly transitions from (near) exponential to inverse power, beyond which the system is unstable. The analysis shows the crucial role of combining more than two clusters in elongating the tail of the size distribution generated by the system and, thus, in increasing the range of validity of the inverse power law. We also point out an interesting connection of the proposed model with Catalan-like integer sequences.

## 1. Introduction

### 1.1. Forest-Fire Models

In the 30 years since simple forest-fire models [1] (FFM) were proposed, including Drossel-Schwabl FFM [2], physicists, mathematicians and other scientists have used the model and its generalizations to understand various properties of complex systems and their relationship to diversified areas of scientific research under their interest. Those investigations are often related to self-organized criticality [3].

Despite its long history, new properties of FFM are still being discovered, such as self-organized bi-stability in 2017 [4] in the Random Domino Automaton (RDA) [5] or self-organized multi-stability in 2021 [6], in Henley FFM [7]. The first is interpreted as possibly meaningful to mega-earthquakes [8], the second as related to ecosystems [9].

The Drossel-Schwabl FFM [2] is remarkable for two main reasons. The first is its role in understanding the spread of fires—although obviously oversimplified, it was found that it reproduces some meaningful properties of real wildfires well [1,10,11,12,13]. The second reason is that, due to its simplicity, it allows analytical approaches to develop explanations of various properties of complex systems [3]. Here, we focus on the second aspect.

### 1.2. Extension of RDA to Bethe Lattice Geometry

In this article, we extend the definition of a system [5], to the geometry of the Bethe lattice (or Cayley tree)—that is, to an infinite graph without loops, each vertex of which connects to an equal number of other vertices. Such regular geometry allows for maintaining a good analytical structure of the one-dimensional model equations, but in contrast to the one-dimensional protoplast and in agreement with realistic situations, it also allows for merging more than two clusters (equivalent to forest patches), which has a significant impact on increasing the size of clusters and, consequently, to a greater spread of fires. The results indicate the key role of this merging in the occurrence of large forest patches or, more precisely, in extending the validity range of the inverse power distribution. In this way, we identify the factor that is responsible for the appearance of inverse power distributions in the model. This mechanism is also present and plays a significant role in the formation of frequently observed inverse power distributions for various phenomena occurring in nature [14].

FF models are usually in the form of probabilistic cellular automata [15] with a given set of parameters. Most of the analytical results available for 1D models are difficult to obtain for higher dimensions, and, thus, 2D models are commonly explored mainly by simulations [3]. One of the main reasons for this, in the case of 2D FF models, is the lack of a universal relationship between the area of an arbitrarily shaped fragment of the forest (determining the chance of ignition by lightning) and its specific shape, including the length of the circumference (which influences the contact of forest patches and, as a result, the possibility of fire transmission).

### 1.3. Connections of RDA with Earthquake Statistics and Integer Sequences

The Random Domino Automaton [5] was formulated as a simple model of earthquake statistics and introduces a generalization of the constant ignition probability parameter to a function of the size of a cluster. This generalization—undoubtedly justified in the context of earthquake statistics (see [16])—also leads to various analytical results for the model itself [4,5] and also to a surprising connection with the field of integer sequences [17]; namely, the equation describing the steady state for the RDA takes [18] the form of the Motzkin number recurrence [19]. Using analogous methods, it is possible to construct systems leading to other Catalan-like recurrences—see [20] and the references therein.

In its original form, the RDA terminology did not refer to trees, forests, and their fires, but rather to balls that group into clusters as they fill adjacent cells, and to avalanches, corresponding to forest fires. In this article, we prefer to retain this original choice.

### 1.4. RDA on Bethe Lattice and Power-Law Distributions

In the article we extend the notion of 1D RDA to the geometry of Bethe lattice [21], i.e., an infinite symmetric regular tree where all vertices have the same number of neighbors. Bethe lattice models are used in statistical mechanics (see, for example, [22,23]), because they can provide useful insight, in spite of rather rough approximation of real interactions due to the lack of cycles.

By introducing an appropriate classification of empty network sites, the set of equations describing the steady state of the system is derived using combinatorial analysis. The special choice of parameters that define the dynamics of the system leads to a solvable reduction of the set of equations.

An important and far-from-obvious property of the system is that inverse power parameters do not lead to scale-free solutions. The obtained cluster-size distributions start with an inverse power-law relationship, and, then, for large values, they have a fast, exponential-like decay. The system generates a natural truncation of power-law distributions. This upper bound of validity is a key characteristic of power-law distributions for natural phenomena [24,25]. This property of the model (in the 1D version) was used to fit the real data of tremors from a copper mine in Poland [16].

The analysis of the solutions shows the key role of combining more than two clusters in shifting to larger values the exponential decay of the cluster-size distribution generated by the system, and thus increasing the range of validity of the inverse power law.

An elongation of the heavy-tail part of the distribution also occurs when the model parameter δ, defining the probability of cluster removal (which is equivalent to forest fire), approaches a boundary value that depends on other model parameters. An important new property of the model defined on the Bethe lattice is that this boundary value corresponds to a “moderate” (isolated from 1) value of the system density. In the case of the one-dimensional model and for special choice of parameters, an inverse power distribution appears in the limit when the density of the system tends to 1 [18]. This is particularly important, since a density close to 1 corresponds to an almost completely filled system, which is not realistic in the context of forest-fire models, nor in many other systems where the model might be applied. Note that inverse power distributions for the “moderate” density may appear in the one-dimensional model, but for significantly different parameters, strongly preferential for the appearance of large clusters [4].

### 1.5. The Plan of the Article

The plan of the article is as follows. In Section 2, we introduce the definition of the model on Bethe lattice and discuss the classification of empty cells into types, which is crucial for the concept. In Section 3, we introduce the basic variables for the model and derive the relationships between them from the model geometry. Section 4 contains derivations of steady-state equations for the density, number of clusters, number of empty clusters, and number of empty cells of all types. We also indicate there the relations between the obtained equations. In Section 5, we reduce the obtained equations to the case of interest, when the probability parameters are constant for clusters, regardless of their size (in the Drossel-Schwabl model, they were constant for the cell). Then, we present two methods for solving the obtained system of seven equations and justify the existence of a range of variability for the parameter δ responsible for avalanches (fires), including the limit related to the inverse power-law distribution for a “regular” (not approaching 1) value of density. We also briefly discuss the recurrences that result from the system under consideration. In Section 6, we demonstrate and discuss with examples the effect of merging two and three clusters on the shape of the cluster-size distribution and discuss the transition to the inverse power-law distribution. Lastly, Section 7 summarizes the results and discusses the perspectives for further research.

## 2. Definition of RDA on the Bethe Lattice

Assume that the automaton cells are placed in nodes of a Bethe lattice, or Cayley tree, that is, a tree (infinite connected cycle-free graph) where each node is connected to the same number *k* of neighbors. The fixed number *k* is called the coordination number of the Bethe lattice. For simplicity, we will present the construction of the automaton and the resulting equations and formulas for the simplest non-trivial case, namely k=3. Transferring the construction for higher coordination numbers is straightforward.

Each cell of the lattice can be in one of two states: empty or occupied (by a ball, say). Thus, occupied cells form clusters of various sizes and shapes. By size of a cluster, we mean the number of occupied cells. Also, for the description of the automaton, it is indispensable to investigate the structure of empty clusters, i.e., clusters formed by empty cells.

The evolution of the automaton is defined as follows. In each time step, one cell of the system is chosen and each cell has the same chance. Thus, the probability of choosing an occupied cell is equal to the fraction of occupied cells, i.e., to the density of the system. Then, the following update of the state of the system is made. If the chosen cell is occupied, then, with probability μ, the cluster containing the cell is removed (its cells become empty). If the chosen cell is empty, then, with probability *c*, the cell becomes occupied. The update procedure is repeated in the next time step.

It is convenient to interpret the evolution rules in terms of the incoming ball triggering an avalanche, rebounding (or scattering) of the incoming ball, and occupation of the cell by the ball, respectively.

In general, the probabilities μ and *c* can depend on arbitrary parameters related to a state of the lattice. A way of assigning values to those probabilities specifies dynamical properties of the automaton. A non-trivial task is to define μ and *c* in a way that enables one to derive equations describing the properties of the automaton.

To achieve this aim, we distinguish four types of empty cells, depending on their neighborhood. If the coordination number is *k*, there are (k+1) types of cells.

- The creating cells—empty cells with all **three neighbors** being **empty**. Their occupation create a new cluster.
- The enlarging cells—empty cells with exactly **one neighbor** being **occupied**, and **two** being **empty**. Their occupation enlarge the adjacent cluster.
- The merging cells of $C_{2}$-type—empty cells with exactly **two neighbors** being **occupied**, and **one** being **empty**. Their occupation merges the two adjacent clusters.
- The merging cells of $C_{3}$-type—empty cells with all **three neighbors** being **occupied**. Their occupation merges the three adjacent clusters.

**Figure 1:**
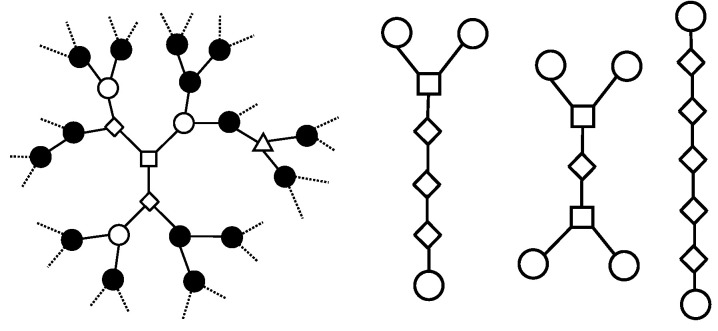
(**Left**) All types of cells of the Random Domino Automaton on the Bethe lattice with coordination number 3—four types of empty cells and one type of occupied cell. Symbols ☐, ◊, ◯, △, • represent creating, enlarging, merging of $C_{2}$-type & $C_{3}$-type, and occupied cells, respectively. (**Right**) Different forms of empty 7-clusters in Bethe lattice for coordination number 3. Note that each cell has three links, and connections to occupied cells are not shown for simplicity.

All four types of cells are presented in Figure 1 for an exemplary state of the automaton.

The probability of triggering an avalanche μ can be defined as an arbitrary function of the size of the cluster that contains the chosen cell. The probability *c* can be given by constants $c_{i}\in \left[0,1\right]$, i=0,1,2,3, for each type of empty cell, where the subscript refers to the number of neighboring occupied sites:

(1) $$c=\left\{\begin{array}{cc} c_{0} & \mathrm{for}\;\mathrm{creating}\;\mathrm{cell},\\ c_{1} & \mathrm{for}\;\mathrm{enlarging}\;\mathrm{cell},\\ c_{2} & \mathrm{for}\;\mathrm{merging}\;\mathrm{cell}\;\mathrm{of}\;C_{2}-\mathrm{type},\\ c_{3} & \mathrm{for}\;\mathrm{merging}\;\mathrm{cell}\;\mathrm{of}\;C_{3}-\mathrm{type}. \end{array}\right.$$

Finally, we recall the following useful property of the Bethe lattice: the relation between the size of a cluster *i* and the number of adjacent empty cells is given by

(2) i(k−2)+2.

This fact can be easily proved by induction with respect to the size *i*.

## 3. Notation and Identities

Consider a finite part of the Bethe lattice that contains a large number *N* of cells. We refer to *N* as the size of the system, and we relate the variables introduced below to this part of the infinite Bethe lattice. We take advantage of the homogeneity of the system—the region under consideration is representative of the entire network. Denote the total number of clusters in this part by *n* and the total number of empty clusters in this part by $n^{0}$. Note that, contrary to the 1-dimensional RDA [5], *n* and $n^{0}$ are not necessarily equal in case of the Bethe lattice. That is because, for example, it is possible to reduce *n* by merging two clusters, keeping $n^{0}$ unchanged, that is, if a cell of $C_{2}$-type is occupied (see Figure 1).

Denote the number of clusters of size *i* by $n_{i}$ and the number of empty clusters of size *i* by $n_{i}^{0}$. Again, contrary to the 1-dimensional RDA, the above notion does not characterize the structure in a complete way. It is necessary to indicate various types of cells for empty clusters. For example, Figure 1 shows all three different structures of empty clusters of size 7. To distinguish between all those forms, we introduce an additional subscript ‘*j*’ which indicates the number of creating cells in the empty *i*-cluster. Remarkably, it characterizes all forms completely, as stated below.

**Fact** **1.** *In the Bethe lattice with coordination number* 3*, for any empty cluster of size i≥2 and the number of creating cells equal to j, the number of merging cells of $C_{2}$-type is equal to (j+2) and the number of enlarging cells is (i−2j−2). The index j varies from* 0 *to $\left\lfloor \frac{i}{2}-1\right\rfloor$. The symbol   denotes the floor function.*

A proof of Fact 1 is presented in the Appendix A. Respective facts could be formulated for other coordination numbers, which is also shown in the Appendix A.

Denote by $n_{i,j}^{0}$ the number of empty clusters of size *i* with *j* creation cells. Then, we have the following formulas. The density ρ of the system is given by

(3) $$\rho =\frac{1}{N}\sum_{i\geq 1}in_{i},$$

the number of clusters *n* of the system is

(4) $$n=\sum_{i\geq 1}n_{i},$$

and the number of empty clusters $n^{0}$ of the system reads

(5) $$n^{0}=\sum_{i\geq 1}n_{i}^{0}=n_{1}^{0}+\sum_{i\geq 2}\sum_{j=0}^{\left\lfloor \frac{i}{2}-1\right\rfloor }n_{i,j}^{0}.$$

It follows that

(6) $$\left(1-\rho \right)=\frac{1}{N}\sum_{i\geq 1}in_{i}^{0}=\frac{1}{N}\left[n_{1}^{0}+\sum_{i\geq 2}\sum_{j=0}^{\left\lfloor \frac{i}{2}-1\right\rfloor }in_{i,j}^{0}\right].$$

Denote by $x_{i}$, i=0,1,2,3 the total number of **empty cells** with *i* neighboring cells being occupied. Thus,

(7) $$x_{0}+x_{1}+x_{2}+x_{3}=\left(1-\rho \right)N.$$

The total number of creating cells $x_{0}$ in the system is

(8) $$x_{0}={\hat{n}}^{0}=\sum_{i\geq 4}\sum_{j=0}^{\left\lfloor \frac{i}{2}-1\right\rfloor }jn_{i,j}^{0}=\sum_{i\geq 1}\sum_{j=0}^{\left\lfloor \frac{i}{2}-1\right\rfloor }jn_{i,j}^{0},$$

where the symbol ${\hat{n}}^{0}$ is introduced. The total number of enlarging cells $x_{1}$ in the system is

(9) $$\begin{array}{cc} x_{1} & =\sum_{i\geq 3}\sum_{j=0}^{\left\lfloor \frac{i}{2}-1\right\rfloor }\left(i-2j-2\right)n_{i,j}^{0}=\left(1-\rho \right)N-2{\hat{n}}^{0}-2n^{0}+n_{1}^{0}. \end{array}$$

The total number of merging cells of $C_{2}$-type $x_{2}$ is

(10) $$\begin{array}{cc} x_{2} & =\sum_{i\geq 2}\sum_{j=0}^{\left\lfloor \frac{i}{2}-1\right\rfloor }\left(j+2\right)n_{i,j}^{0}={\hat{n}}^{0}+2\left[\sum_{i\geq 1}\sum_{j=0}^{\left\lfloor \frac{i}{2}-1\right\rfloor }n_{i,j}^{0}-n_{1}^{0}\right]={\hat{n}}^{0}+2n^{0}-2n_{1}^{0}. \end{array}$$

The total number of merging cells of $C_{3}$-type $x_{3}$ is nothing but the total number of empty clusters of size 1, so

(11) $$x_{3}=n_{1}^{0}.$$

Any four out of the five Equations (7)–(11) define a change of variables:

(12) $$\left\{x_{0},x_{1},x_{2},x_{3}\right\}\;⟷\;\left\{n^{0},n_{1}^{0},{\hat{n}}^{0},\rho \right\}.$$

Having formulas for $x_{i}$s as functions of the parameters $\left\{\mu ,c_{i},N\right\}$, it is straightforward to find remaining quantities describing the stationary state for the automaton; for example,

(13) $$n^{0}=\frac{1}{2}\left(x_{2}+2x_{3}-x_{0}\right).$$

**Remark** **1.** *Since $n^{0}>n_{1}^{0}$, the following condition must hold:*

(14) $$x_{2}>x_{0}.$$

## 4. Balance Equations for RDA on the Bethe Lattice of Coordination Number 3

We assume a specific set of parameters is given and that the automaton is in a stationary state. In order to derive respective equations, we use the following condition: for a given quantity (such as density, number of clusters, or number of cells of specific type), the probabilities of increase and decrease in the quantity balance each other. In other words, expected values of flow-in and flow-out are equal. This approach is based on finding a fixed point of the system’s evolution equations.

A more rigorous approach to justifying the derived equations is based on the Markov chain theory. For the definition of RDA in terms of Markov chains, see [26]. The justification for the existence of the statistically stationary state in this context is explained in [27].

### 4.1. Balance Equation for ρ

In a single time step, the number of occupied cells may remain unchanged (when the new ball is reflected) or may increase by one (when the chosen empty cell becomes occupied) or decrease by *i* (when the avalanche of size *i* is triggered).

*Gains:* All types of empty cells may become occupied; thus, contributions are just products of probabilities that the given cell is chosen times *c* according to Formula (1).

*Losses:* The probability of relaxation of a cluster of size *i* is $\mu_{i}\left(in_{i}/N\right)$ and any possible size *i* contributes.

Thus, the balance equation for the density ρ in the stationary state reads

(15) $$c_{0}x_{0}+c_{1}x_{1}+c_{2}x_{2}+c_{3}x_{3}=\sum_{i\geq 1}\mu_{i}n_{i}i^{2}.$$

### 4.2. Balance Equation for N

*Gains:* The number of clusters may increase (by 1 in a single time step) only if a new cluster is created, i.e., a creating cell becomes occupied.

*Losses:* The number of clusters decreases by merging two or three clusters and by triggering an avalanche.

Hence, the balance equation for the total number of clusters *n* in stationary state is

(16) $$c_{0}x_{0}-c_{2}x_{2}-2c_{3}x_{3}=\sum_{i\geq 1}\mu_{i}n_{i}i.$$

### 4.3. Balance Equation for $N^{0}$

*Gains:* The number of empty clusters can increase as a result of “cutting” an empty cluster into pieces, i.e., by occupation of creating or enlarging cells. The increase is by 2 and by 1, respectively.

*Losses:* The occupation of a cluster of size 1 (a merging cell of $C_{3}$-type) reduces the $n^{0}$ by 1. Another reduction of $n^{0}$ is by triggering an avalanche. According to Fact 1, the number of empty cells in the perimeter of *i*-cluster is (i+2), and the removal of the *i*-cluster results in the merging of i+2 empty clusters into one; hence, $n^{0}$ is reduced by (i+1).

Thus, the balance equation for the total number of empty clusters is

(17) $$2c_{0}x_{0}+c_{1}x_{1}-c_{3}x_{3}=\sum_{i\geq 1}\mu_{i}n_{i}i\left(i+1\right).$$

**Remark** **2.** *There is a dependency relation between the balance Equations (15)–(17), namely Equation (ρ)= Equation$(n^{0})\;-$ Equation(n).*

This relation is a consequence of the property expressed in Fact 1, and may be viewed as an analogue of equation $n=n^{0}$ valid for a one-dimensional RDA.

**Remark** **3.** *Note that Equation (13) gives $x_{2}+2x_{3}-x_{0}>0$, while, from Equation (16), it follows that $c_{2}x_{2}+2c_{3}x_{3}-c_{0}x_{0}<0$. This implies restrictions on the choice of parameters $c_{i}$ in order to obtain a stationary state. In particular, it implies that*

(18) $$c_{0}>\min\left\{c_{2},c_{3}\right\},$$

*and thus excludes the case $c_{0}=c_{2}=c_{3}$.*

Violating the condition (18) causes merging to outweigh the formation of new clusters (of size 1) to such a significant extent that it prevents reaching a steady state due to the decrease in the number of clusters (see also Equation (33) below). If the probabilities of inducing an avalanche for large clusters are sufficiently small, this situation leads to the formation of clusters of increasingly larger sizes. An analogous situation in the case of finite one-dimensional RDA was described using Markov chains in [26].

### 4.4. Approximations

The balance equations for ρ, *n*, $n^{0}$ derived above are exact. Next, we derive the balance equations for $x_{i}$’s and for $n_{i}$’s. To this aim, we use the following approximations.

There is no correlation between sizes of adjacent clusters and empty clusters, which is the mean field approximation. There is no correlation between empty cells of various types, and, thus, the probability *T* of occupation of an empty cell adjacent to another empty cell can be expressed as

(19) $$\;T=\sum_{i=0}^{2}\frac{c_{i}x_{i}}{x_{0}+x_{1}+x_{2}}.$$

To refer to various transitions between cells in a transparent way, we use the following notion. By $\left(C_{1}\to C_{0}\right)$, we refer to a transition of an enlarging cell into a creating cell, and by $\left(•\to C_{0}\right)$, we refer to a transition of an occupied cell to a creating cell, and analogously for other transitions.

### 4.5. Equations for the Numbers of Empty Cells

Taking into account the following transitions, for gains $\left(•\to C_{0}\right)$ and $\left(C_{1}\to C_{0}\right)$, and for losses $\left(C_{0}\to •\right)$ and $\left(C_{0}\to C_{1}\right)$, we arrive at the equation

(20) $$\left(c_{0}+3T\right)x_{0}=\sum_{i\geq 1}\mu_{i}n_{i}i^{2}+\frac{x_{1}}{n}\sum_{i\geq 1}\mu_{i}n_{i}i.$$

The transitions for gains $\left(C_{0}\to C_{1}\right)$ and $\left(C_{2}\to C_{1}\right)$, and for losses $\left(C_{1}\to •\right)$, $\left(C_{1}\to C_{0}\right)$ and $\left(C_{1}\to C_{2}\right)$, lead to the equation

(21) $$3Tx_{0}-\left(2T+c_{1}\right)x_{1}=\frac{x_{1}-2x_{2}}{n}\sum_{i\geq 1}\mu_{i}n_{i}i.$$

The transitions for gains $\left(C_{1}\to C_{2}\right)$ and $\left(C_{3}\to C_{2}\right)$ and for losses $\left(C_{2}\to •\right)$, $\left(C_{2}\to C_{1}\right)$ and $\left(C_{2}\to C_{3}\right)$ lead to

(22) $$2Tx_{1}-\left(T+c_{2}\right)x_{2}=\frac{2x_{2}-3x_{3}}{n}\sum_{i\geq 1}\mu_{i}n_{i}i.$$

Finally, the following transitions for gains $\left(C_{2}\to C_{3}\right)$ and for losses $\left(C_{3}\to •\right)$ and $\left(C_{3}\to C_{2}\right)$ give

(23) $$Tx_{2}=x_{3}\left(c_{3}+\frac{3}{n}\sum_{i\geq 1}\mu_{i}n_{i}i\right).$$

**Remark** **4.** *The following relation between Equations (20)–(23) and (15) is satisfied: $\sum_{i=0}^{3}$$\text{Equation}\left(x_{i}\right)=\text{Equation}\left(\rho \right)$. Note that the identity is satisfied for any form of T. It shows that the approximations described in Section 4.4 are consistent with the exact Equation (15) for density ρ.*

### 4.6. Equations for the Distribution of Clusters $N_{i}$

Similarly, considering gains and losses for clusters of given size *i*, one can arrive at the following balance equations for $n_{i}$’s:

(24) $$\begin{array}{cc} n_{1} & =\frac{1}{\mu_{1}+Y}\left(c_{0}x_{0}\right), \end{array}$$

(25) $$\begin{array}{cc} n_{2} & =\frac{1}{2\mu_{2}+Y}\left(c_{1}x_{1}\frac{n_{1}}{n}\right), \end{array}$$

(26) $$\begin{array}{cc} n_{3} & =\frac{1}{3\mu_{3}+Y}\left(c_{1}x_{1}\frac{n_{2}}{n}+c_{2}x_{2}{\left(\frac{n_{1}}{n}\right)}^{2}\right), \end{array}$$

(27) $$\begin{array}{cc} n_{i} & =\frac{1}{i\mu_{i}+Y}\left(c_{1}x_{1}\frac{n_{i-1}}{n}+c_{2}x_{2}\sum_{k=1}^{i-2}\frac{n_{k}}{n}\frac{n_{i-k-1}}{n}+c_{3}x_{3}\sum_{k=1}^{\left(i-3\right)}\sum_{l=1}^{\left(i-k-2\right)}\frac{n_{k}}{n}\frac{n_{l}}{n}\frac{n_{i-k-l-1}}{n}\right), \end{array}$$

for i≥4, and where

(28) $$Y=\frac{1}{n}\left(c_{1}x_{1}+2c_{2}x_{2}+3c_{3}x_{3}\right).$$

The set of equations above is in the form of cascade equations, i.e., it enables the calculation of $n_{i}$, having calculated $n_{i-1},n_{i-2},\ldots ,n_{1}$, for known values of other parameters.

**Remark** **5.** *Equations (24)–(27) sum up to the balance equation for ρ (15). It shows that the approximations described in Section 4.4 are consistent with the exact Equation (15).*

**Remark** **6.** *Equations (24)–(27) indicates a way of generalization for higher coordination numbers.*

**Remark** **7.** *Note that Equations (24)–(27) can be solved for parameters $\mu_{i}$ in terms of $c_{i}$s, $x_{i}$s. It opens the possibility of finding parameters $\mu_{i}$ that produce a given distribution $n_{i}$. This one-to-one correspondence for 1D RDA is developed in [5,16].*

## 5. The Solution for a Special Case

Up to this point, we have considered a general version of RDA with arbitrary probabilities $\mu_{i}$. This large number of parameters gives the model enormous flexibility. This property was exploited, for example, in the case of one-dimensional RDA when fitting to real measurement data on the magnitude distribution of earthquakes in a copper mine [16].

In this section, we strongly restrict this freedom by choosing the following form:

(29) $$\mu_{i}=\frac{\delta }{i},$$

where δ is a constant, and δ∈[0,1]. This choice sets the probability of removing a cluster from the system regardless of its size.

### 5.1. The Set of Equations

The form of parameter μ, given by Formula (29), leads to significant simplification of the system’s equations. In particular, we have

(30) $$\sum_{i\geq 1}\mu_{i}n_{i}i=\delta n\;\mathrm{and}\;\sum_{i\geq 1}\mu_{i}n_{i}i^{2}=\delta \rho N,$$

and we can eliminate these terms from Equations (20)–(23) by using Equations (15)–(17).

In view of Remark 2, we skip Equation (17), and in view of Remark 4, we skip Equation (22). Thus, the complete set of equations for the six variables $\left\{x_{i},\rho ,n\right\}$ is

(31) $$\begin{array}{ccc} x_{0}+x_{1}+x_{2}+x_{3} & = & (1-\rho )N, \end{array}$$

(32) $$\begin{array}{ccc} c_{0}x_{0}+c_{1}x_{1}+c_{2}x_{2}+c_{3}x_{3} & = & \delta \rho N, \end{array}$$

(33) $$\begin{array}{ccc} c_{0}x_{0}-c_{2}x_{2}-2c_{3}x_{3} & = & \delta n, \end{array}$$

(34) $$\begin{array}{ccc} \left(c_{0}+3T\right)x_{0} & = & \delta \rho N+\delta x_{1}, \end{array}$$

(35) $$\begin{array}{ccc} 3Tx_{0}-\left(2T+c_{1}\right)x_{1} & = & \delta (x_{1}-2x_{2}), \end{array}$$

(36) $$\begin{array}{ccc} Tx_{2}-c_{3}x_{3} & = & 3\delta x_{3}, \end{array}$$

where *T*, defined in Equation (19), gives the nonlinearity to these equations. It is assured in Appendix B that the above has at most one physically admissible solution. Hence, multi-stabilites cannot be observed in this system. The condition for the existence of the solution, i.e., the existence of the stable state, is investigated in the following subsections.

**Remark** **8.** *From Equations (31) and (32), it follows that*

(37) $$<c>:=\frac{c_{0}x_{0}+c_{1}x_{1}+c_{2}x_{2}+c_{3}x_{3}}{x_{0}+x_{1}+x_{2}+x_{3}}=\frac{\delta \rho }{\left(1-\rho \right)},$$

*and thus*

(38) $$\min_{i\in \{0,1,2,3\}}\left\{c_{i}\right\}<\frac{\delta \rho }{\left(1-\rho \right)}<\max_{i\in \{0,1,2,3\}}\left\{c_{i}\right\}\leq 1.$$

*Thus, we obtain upper and lower bounds to the density value.*

In particular,

(39) $$\rho <\frac{1}{1+\delta }.$$

The respective condition for 1D RDA is $\rho ={\left(1+\delta \right)}^{-1}$ (compare [5]).

**Remark** **9.** *From Equations (32) and (33) and the definition of Y (Equation (28)), it follows that*

(40) Y=δ(<i>−1),

*where <i> is the average size of a cluster, which means that Y/δ is a measure of how much the average size of a cluster exceeds the minimal value* 1.

### 5.2. The Solution Procedure

First, we calculate $Tx_{0}$, $Tx_{1}$, and $Tx_{2}$ from Equations (34)–(36),

(41) $$\begin{array}{ccc} 3Tx_{0} & = & \delta \rho N+\delta x_{1}-c_{0}x_{0}, \end{array}$$

(42) $$\begin{array}{ccc} 2Tx_{1} & = & \left(c_{2}+2\delta \right)x_{2}+c_{3}x_{3}, \end{array}$$

(43) $$\begin{array}{ccc} Tx_{2} & = & \left(3\delta +c_{3}\right)x_{3}. \end{array}$$

Then we substitute them into Equation (19) and eliminate δρN using Equation (32), and thus, we obtain the linear equation for $x_{0}$, $x_{1}$, $x_{2}$, and $x_{3}$ only. Together with Equations (31)–(33), it forms a set of four linear equations.

The solution of the set is as follows:

(44) $$x_{i}=\alpha_{i}^{\left(n\right)}n+\alpha_{i}^{\left(\rho N\right)}\rho N+\alpha_{i}^{\left(N\right)}N\;\mathrm{for}\;i=0,1,2,3,$$

where coefficients $\alpha_{i}^{\left(\cdot \right)}$ depend on parameters $c_{i}$ and δ only (the explicit expressions are given in Appendix C).

Next, from Equation (43), we extract *T* and substitute it into Equations (41) and (42), which, after substitution of solutions given by Equation (44), are just two ellipsoids:

(45) $$\begin{array}{ccc} p^{\left(nn\right)}n^{2}+p^{\left(n\rho \right)}n\rho +p^{\left(\rho \rho \right)}\rho^{2}+p^{\left(n\right)}n+p^{\left(\rho \right)}\rho +p^{\left(1\right)} & = & 0, \end{array}$$

(46) $$\begin{array}{ccc} q^{\left(nn\right)}n^{2}+q^{\left(n\rho \right)}n\rho +q^{\left(\rho \rho \right)}\rho^{2}+q^{\left(n\right)}n+q^{\left(\rho \right)}\rho +q^{\left(1\right)} & = & 0, \end{array}$$

where coefficients $p^{\cdot }$ and $q^{\cdot }$ depend on the parameters $c_{i}$ and δ only. The intersections of those ellipses define the solutions (n,ρ), if n>0, ρ>0, and if the condition given by inequality (14) is satisfied. In fact, the inequality (14) is just a linear condition:

(47) $$s^{\left(n\right)}n+s^{\left(\rho \right)}\rho +s^{\left(1\right)}>0,$$

where coefficients $s^{\left(\cdot \right)}$ depend only on parameters $c_{i}$ and δ.

### 5.3. The Range of δ

For given parameters $c_{0},c_{1},c_{2},c_{3}$, it is possible to calculate a range of $\delta \in \left(\delta_{min},\delta_{max}\right)\subset \left(0,1\right)$, for which the system is in stationary state and the respective solution exists.

**Figure 2:**
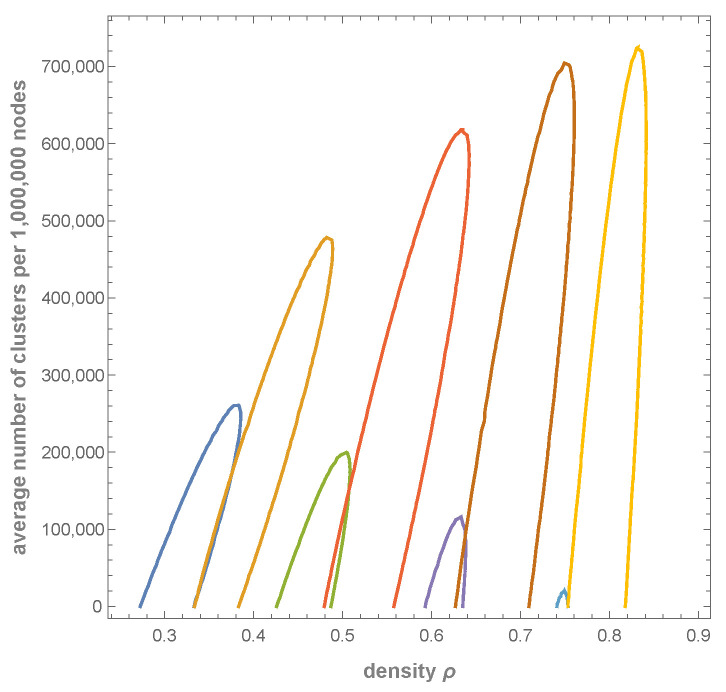
Condition n=0 gives the value of $\delta_{min}$. Pairs of ellipses are given by Equations (45) and (46) for $\delta =1,\frac{1}{2},\frac{1}{4},\frac{1}{8}$, respectively, starting from the left. The valid solution (ρ,n) is given by intersection, where n>0. The rightmost case is close to $\delta_{min}=0.121105$, corresponding to maximal density ρ=0.75779. Parameters $c_{i}$ are $c_{0}=1,c_{1}=0.5,c_{2}=0.25,c_{3}=0.125$ (see also Table 2). The slight irregularities of the ellipses are due to numerical errors.

Depending on the value of the parameter δ (in relation to the other parameters), the intensity of cluster removal changes. In the case of a low value, the merging dominates and the number of clusters decreases. So, Equations (45) and (46) and the additional condition n=0 give the minimal value $\delta_{min}$. This situation is illustrated in Figure 2.

**Figure 3:**
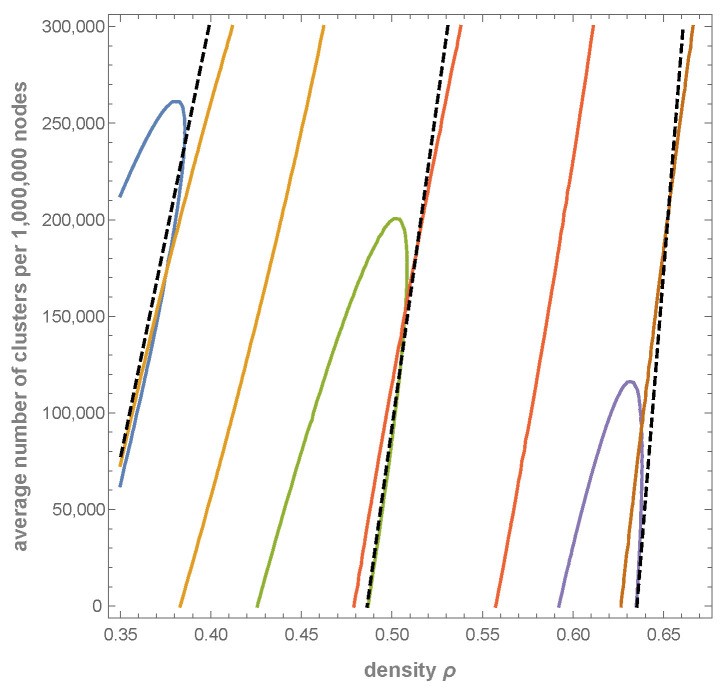
Inequality (47) gives the value of $\delta_{max}$. Pairs of ellipses given by Equations (45) and (46) and dashed lines given by condition (47) for $\delta =1,\frac{1}{2},\frac{1}{4}$, respectively, starting from the left. The valid solution (ρ,n) is given by intersection on the left from the dashed line. The leftmost intersection is *not* a valid solution; it violates condition (47). Two other intersections are valid. The boundary is for $\delta_{max}=0.606063$, which corresponds to n= 164,108, and ρ=0.470532. Parameters $c_{i}$ are $c_{0}=1,c_{1}=0.5,c_{2}=0.25,c_{3}=0.125$ (see also Table 2).

On the other hand, high values of δ lead to an increase in the number of clusters and a simultaneous decrease in the number of empty clusters with size larger than 1. The corresponding value of $\delta_{max}$ is obtained from Equations (45) and (46) and the condition $x_{2}=x_{0}$ (see Equation (14)) which defines a border of a half-plane (see inequality (47)). This situation is illustrated in Figure 3.

To see in detail the quantitative changes of various parameters of the system for different values of δ, we consider the relevant example in Section 6.2.

### 5.4. Recurrences

For $c_{2}=c_{3}=0$, when only enlarging takes place, the solution of Equations (24)–(27) is given by geometric series

(48) $$\frac{n_{i}}{n}=\frac{1}{\delta +Y}\frac{c_{0}x_{0}}{n}{\left(\frac{1}{\delta +Y}\frac{c_{1}x_{1}}{n}\right)}^{i-1}\;\mathrm{for}\;i=1,2,\ldots$$

If $c_{3}=0$, then Equations (24)–(27) are

(49) $$\begin{array}{ccc} \frac{n_{1}}{n} & = & \frac{1}{\delta +Y}\frac{c_{0}x_{0}}{n}, \end{array}$$

(50) $$\begin{array}{ccc} \frac{n_{i}}{n} & = & \frac{1}{\delta +Y}\left(\frac{c_{1}x_{1}}{n}\frac{n_{i-1}}{n}+\frac{c_{2}x_{2}}{n}\sum_{k=1}^{i-2}\frac{n_{k}}{n}\frac{n_{i-1-k}}{n}\right), \end{array}$$

where i=2,3,…. Then, rescaling of variables $n_{i}$ to $M_{i}$ by the following formula

(51) $$\frac{n_{i}}{n}=\frac{c_{1}x_{1}}{c_{2}x_{2}}{\left(\frac{c_{1}x_{1}}{(\delta +Y)n}\right)}^{i}M_{i},$$

it follows from direct calculation that Equations (49)–(50) become

(52) $$\begin{array}{ccc} M_{1} & = & M_{2}=\frac{\left(c_{0}x_{0}\right)\left(c_{2}x_{2}\right)}{{\left(c_{1}x_{1}\right)}^{2}} \end{array}$$

(53) $$\begin{array}{ccc} M_{i} & = & M_{i-1}+\sum_{k=1}^{i-2}M_{k}M_{i-1-k}\;\mathrm{for}\;i=3,4,\ldots \end{array}$$

which is the form of the Motzkin numbers recurrence [18,19], except for the initial values.

To construct an (asymptotically) inverse power solution that corresponds to Motzkin numbers, we require $M_{1}=M_{2}=1$; thus, $c_{1}x_{1}=\sqrt{\left(c_{0}x_{0}\right)\left(c_{2}x_{2}\right)}$. Next, since Motzkin numbers have asymptotics $M_{i}∼3^{i}i^{-\frac{3}{2}}$, then, in order to compensate the exponential growth in Equation (51), we require $\left(\frac{c_{1}x_{1}}{(\delta +Y)n}\right)=\frac{1}{3}$. Together with Equation (33), it gives $c_{0}x_{0}=c_{2}x_{2}=c_{1}x_{1}=\frac{1}{3}\delta \rho N$, and, in consequence, from Equation (32), it follows that δn=0, i.e., that for the inverse power solution, the number of clusters n⟶0.

For general values of parameters $c_{i}$, Equations (24)–(27) rewritten in terms of $M_{i}$ (given by Equation (51)) take the form

(54) $$\begin{array}{ccc} M_{1} & = & M_{2}=\frac{\left(c_{0}x_{0}\right)\left(c_{2}x_{2}\right)}{{\left(c_{1}x_{1}\right)}^{2}} \end{array}$$

(55) $$\begin{array}{ccc} M_{3} & = & \frac{\left(c_{0}x_{0}\right)\left(c_{2}x_{2}\right)}{{\left(c_{1}x_{1}\right)}^{2}}+{\left(\frac{\left(c_{0}x_{0}\right)\left(c_{2}x_{2}\right)}{{\left(c_{1}x_{1}\right)}^{2}}\right)}^{2} \end{array}$$

(56) $$\begin{array}{ccc} M_{i} & = & M_{i-1}+\sum_{k=1}^{i-2}M_{k}M_{i-1-k}+\frac{\left(c_{1}x_{1}\right)\left(c_{3}x_{3}\right)}{{\left(c_{2}x_{2}\right)}^{2}}\sum_{k=1}^{\left(i-3\right)}\sum_{l=1}^{\left(i-k-2\right)}M_{k}M_{l}M_{i-k-l-1}, \end{array}$$

where i=4,5,….

We may require that the coefficient in Equation (56) is equal to 1, and, thus, we derived generalized Motzkin-type number recurrences related to coordination number 3 of the Bethe lattice. It is the sequence A036765, 1, 1, 2, 5, 13, 36, 104, 309, 939, 2905, 9118, 28,964, 92,940, …, listed in The On-line Encyclopedia of Integer Sequences (OEIS), which gives the number of ordered rooted trees with *i* non-root nodes and all outdegrees smaller or equal to 3 [28]. The condition $M_{1}=M_{2}=1$ leads to $c_{0}x_{0}=c_{2}x_{2}=c_{1}x_{1}=c_{3}x_{3}=\frac{1}{4}\delta \rho N$. But this contradicts Equation (52) for δn, and, thus, such initial conditions cannot be set.

In an analogous way, one may define recurrences for higher coordination numbers.

## 6. Examples

### 6.1. Influence of Merging of Two and Three Clusters

To illustrate the influence of merging of two and three clusters, we start from a system with the following parameters:

$$c_{0}=1\;\mathrm{and}\;c_{1}=c_{2}=c_{3}=0.5,$$

which leads to the following range of δ:

δ∈(0.393177,0.507752).

Then, we choose a value of δ=0.4 close to the inverse power bond, and a value δ=0.5 close to the exponential bond.

Next, for both δ=0.4 and δ=0.5, we set $c_{1}=0.5$ and choose all four combinations of parameters $c_{2},c_{3}\in \left\{0,0.5\right\}$ to present the influence of terms describing the merging of two and three clusters; choosing $c_{i}=0$, the respective merging is turned off. Table 1 contains a variety of parameters for all these cases. In particular, $x_{i}/\left(1-\rho \right)N$ is a fraction of empty cells that are of $C_{i}$-type, <i> is the average size of the clusters, $<i^{0}>$ is the average size of the empty clusters, and $<i^{0}{>}_{2+}$ is the average size of the empty clusters excluding the clusters of size one, i.e.,

(57) $$<i^{0}{>}_{2+}=\frac{\sum_{i\geq 2}n_{i}^{0}i}{\sum_{i\geq 2}n_{i}^{0}}=\frac{<i^{0}>n^{0}-n_{1}^{0}}{n^{0}-n_{1}^{0}}.$$

**Figure 4:**
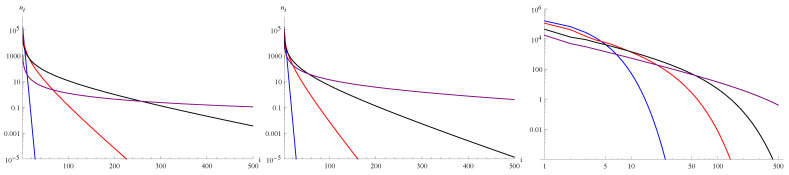
Influence of merging on distribution of $n_{i}$’s calculated from Equations (24)–(27) presented on Lin-Log and Log-Log plots. Parameters $\mu_{i}=\frac{\delta }{i}$, $c_{0}=1,c_{1}=0.5$, δ=0.4 close to the inverse power bond (**left**) and δ=0.5 close to the exponential bond (**middle** and **right**), $N=10^{6}$ and four cases: $c_{2}=0,c_{3}=0$—blue (the steepest curve), $c_{2}=0,c_{3}=0.5$—red, $c_{2}=0.5,c_{3}=0$—black, $c_{2}=0.5,c_{3}=0.5$—purple (the most horizontal). The straight sections of the purple function graph in the rightmost Log-Log plot coincides with an inverse power function with the exponent −1.5.

Figure 4 shows the distributions $n_{i}$ for δ=0.4 (left) and δ=0.5 (middle and right). For both cases, merging of two clusters only (black curve) elongates the tail more than merging of three clusters only (red curve). However, merging two and three clusters simultaneously leads to a much more effective extension of the inverse power part of the plot. This effect is clearly visible even for the parameter δ=0.5 close to the exponential bound.

Merging increases the density. For $c_{3}\neq 0$, the number $n_{1}^{0}$ of empty clusters of size 1 is reduced, as they may become occupied. In addition, it significantly increases the average size of empty clusters $<i_{0}>$.

For the case δ=0.5, close to the exponential bound $\delta_{max}$, it can be seen, from the lowest line of the Table 1, that most empty cells belong to a relatively small number of 517 empty clusters of size larger than 1 (the average size of them is ≈773), and less than 10% of empty cells form almost 40000 clusters of size one. It should also be noted that the number of clusters and the number of empty clusters are almost the same $n\approx n^{0}$. Note that condition $n=n^{0}$ gives δ≈0.499626, which is very close to considered δ=0.5. (The value of δ is calculated from the condition $n=n^{0}$ in the same way as $\delta_{min}$ and $\delta_{max}$).

The lowest line of the upper part (δ=0.4) of Table 1 shows the case close to the inverse power bound $\delta_{min}$ for which the number of clusters is relatively small, and their average size is relatively large. Again, the vast majority of empty clusters are of size 1 and the majority of empty cells belong to empty clusters of sizes larger than 1. The average size of the cluster <i> is ≈128, which is almost 12 times greater than for the respective case with δ=0.5.

### 6.2. Approaching Inverse Power Distribution

To show the smooth transition from exponential bound to inverse power bound, we set the parameters as

$$c_{0}=1,\;c_{1}=0.5,\;c_{2}=0.25,\;c_{3}=0.125.$$

The respective range of δ is

δ∈(0.121105,0.606063).

**Figure 5:**
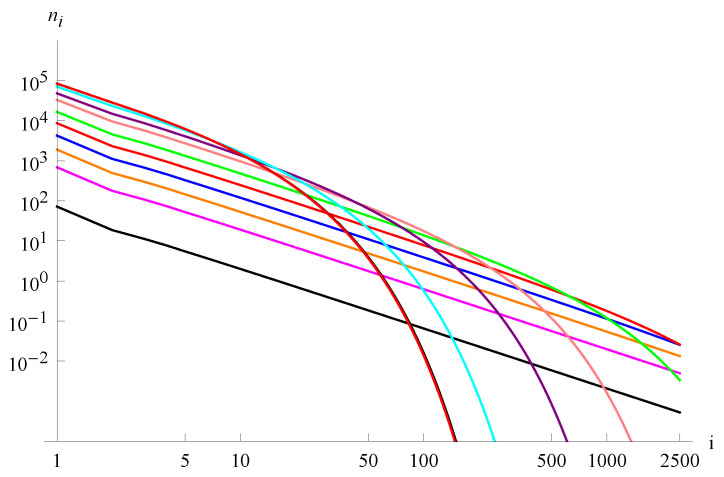
Distribution of $n_{i}$’s calculated from Equations (24)–(27) by choosing $\mu_{i}=\frac{\delta }{i}$ and the parameters as $c_{0}=1,c_{1}=0.5,c_{2}=0.25,c_{3}=0.125,N=10^{6}$ for δ= 0.606, 0.6, 0.44, 0.28, 0.2, 0.16, 0.14, 0.13, 0.125, 0.1225, 0.12125, starting from the top on the left side. Curves for δ=0.606 and 0.6 nearly overlap. The straight sections of the function graphs coincide with an inverse power function with the exponent −1.5.

Thus, we select the following sequence of values: δ as 0.606, 0.6, 0.44, 0.28, 0.2, 0.16, 0.14, 0.13, 0.125, 0.1225, 0.12125, and present respective parameters in Table 2 and in Figure 5. Moreover, we also calculated that $n=n^{0}$ for parameter δ≈0.256404.

In the case presented in Figure 5, the inverse power part can be determined by the observation that n(5)≈10 and n(500)≈1/100; hence, n(i) is of the form $n\left(i\right)\approx 10{\left(\frac{5}{i}\right)}^{\frac{3}{2}}$.

Although the dependence of density ρ and number of clusters *n* is monotonic with changing value of δ, the number of empty clusters $n^{0}$ initially increases with increasing δ and then decreases. As can be seen in Table 2, the comparison of values δ=0.1225 and δ=0.2, for which $n^{0}\approx$ 98,000, the proportions of empty cells of various types are different—the contribution of merging cells of $C_{3}$-type is significantly higher for δ=0.1225, in contrast to the other types of empty cells, for which the contributions are greater for δ=0.2. This is in accordance with the decrease in system density ρ and the average cluster size <i>, as well as with the increase in the average size of empty clusters $<i^{0}>$.

Decreasing the value of the parameter δ leads to an extension of the inverse power part of the cluster-size distribution. In the limit $\delta =\delta_{min}$, the dependence is strictly inverse power. After exceeding this value, the system does not tend to a steady state, and a strong growth of large clusters leads to the instability of the system and consequently to a quasi-periodic change in the density of the system (see [26]). Achieving an inverse power distribution on the verge of instability is a characteristic property of self-organized criticality, and in the presented model, this aspect results directly from the system equations.

## 7. Conclusions

We demonstrate how to transfer the rules defining the Random Domino Automaton to the case of Bethe lattice geometry. This geometry preserves the model’s good mathematical properties, including its solvability for a special choice of its parameters, while also allowing for the consideration of merging of a larger number of clusters (depending on the coordinate number).

To derive equations describing the steady-state system defined on the Bethe lattice, it was necessary to distinguish between different kinds of empty sites, depending on the number of occupied neighbors. This allowed us to present a general method for deriving equations for the Bethe lattice with any coordinate number k. In this paper, detailed calculations were performed for the lowest non-trivial value of k = 3 (The case k = 2 corresponds to the 1D RDA). Empty sites are not only important because they can become occupied, but they are crucial in determining how many clusters can connect in a given place. Merging more than two clusters is a new aspect that we can analytically investigate in the proposed model.

The RDA on the Bethe lattice is a solvable model. We analyze in detail the special case, where the probability of cluster removal is independent of its size. We presented two methods for solving the system of equations that describe the stationary state. Analysis of the equations shows that by changing the parameter δ responsible for the removal of the clusters, we can smoothly transition from a (near) exponential distribution of cluster sizes to an inverse power one. We show how to determine the range of variation of the parameter δ, beyond which the system is unstable (the conditions for the formation of a stationary state are not met). Of particular interest is the state for $\delta ⟶\delta_{min}$ when the tail of the distribution lengthens, reaching the limit of the inverse power distribution. The possibility of combining three clusters means that such a limiting state can be reached for densities isolated from 1, i.e., without completely filling the system, as is the case in the one-dimensional system.

It is worth mentioning here that RDA leads to inverse power-law solutions, unlike the DS model, as shown by systematic simulations in [29]. The main reason for this seems to be the specific choice of parameters $\mu_{i}$ given by (29). An interesting question is whether this modification can also lead to genuine scaling laws for the DS model.

The cluster merging mechanism extends the tail of the cluster-size distribution and has not been previously studied in this context [30]. This is a microscopic mechanism that may be responsible for the common occurrence of long-tailed distributions [14,24,25] for various phenomena shaped by growth and merging processes.

The methods of system analysis presented in this article can be widely applied. A similar approach, though with a different purpose, has recently been demonstrated, for example, in ecological modeling [13] and in the study of tree size distribution [31].

We also mention that the presented model, being a dynamical system on a Bethe lattice, leads to new generalized Catalan-like recurrences—this mathematical aspect, however, is beyond the scope of this paper, and a detailed analysis of its properties will be carried out in another work.

## Figures and Tables

**Table 1:** Parameters corresponding to plots presented in Figure 4. Values of *n*, $n^{0}$ and $n_{1}^{0}$ are expressed per $10^{6}$ cells. In all cases, $c_{0}=1$ and $c_{1}=0.5$.

$c_{2}$	$c_{3}$	δ	ρ	n	$n^{0}$	$n_{1}^{0}$	$\frac{x_{0}}{(1-\rho )N}$	$\frac{x_{1}}{(1-\rho )N}$	$\frac{x_{2}}{(1-\rho )N}$	$\frac{x_{3}}{(1-\rho )N}$	〈i〉	$\langle i^{0}\rangle$	${\langle i^{0}\rangle }_{2+}$
0	0	0.4	0.486071	282,836	94,017	62,962	22.0%	31.6%	34.1%	12.3%	1.82	5.47	14.52
0	0.5	0.4	0.535833	188,651	58,676	40,925	25.1%	33.4%	32.7%	8.8%	2.46	7.91	23.84
0.5	0	0.4	0.572665	80,101	85,192	68,729	22.6%	31.0%	30.3%	16.0%	5.35	5.03	21.87
0.5	0.5	0.4	0.611689	3026	49,310	43,122	26.0%	33.7%	29.2%	11.1%	128.34	7.87	55.78
0	0	0.5	0.454748	268,644	71,715	52,926	24.6%	34.1%	31.5%	9.7%	2.03	7.60	26.20
0	0.5	0.5	0.491362	200,036	45,848	37,211	27.0%	35.3%	30.4%	7.3%	2.54	11.09	54.58
0.5	0	0.5	0.529656	99,161	67,423	58,232	25.0%	33.7%	28.9%	12.4%	4.74	6.98	44.84
0.5	0.5	0.5	0.560624	40,520	40,364	39,847	27.6%	35.5%	27.8%	9.1%	10.84	10.89	772.79

**Table 2:** Values of all variables for parameters $c_{0}=1,c_{1}=0.5,c_{2}=0.25,c_{3}=0.125$ corresponding to Figure 5. Values of *n*, $n^{0}$ and $n_{1}^{0}$ are given per $10^{6}$ cells.

δ	ρ	n	$n^{0}\;$	$n_{1}^{0}$	$\frac{x_{0}}{(1-\rho )N}$	$\frac{x_{1}}{(1-\rho )N}$	$\frac{x_{2}}{(1-\rho )N}$	$\frac{x_{3}}{(1-\rho )N}$	〈i〉	$\langle i^{0}\rangle$	${\langle i^{0}\rangle }_{2+}$
0.12125	0.757612	170	97,886	78,924	16.1%	19.6%	31.7%	32.6%	1428.71	2.48	8.62
0.1225	0.756087	1619	98,078	78,976	16.1%	19.7%	31.8%	32.4%	150.68	2.49	8.63
0.125	0.753067	4468	98,441	79,065	16.2%	19.9%	31.9%	32.0%	55.27	2.51	8.66
0.13	0.747144	9975	99,093	79,192	16.4%	20.2%	32.1%	31.3%	25.30	2.55	8.73
0.14	0.735742	20,277	100,122	79,259	16.7%	20.8%	32.4%	30.0%	13.03	2.64	8.87
0.16	0.714519	38,428	101,253	78,807	17.3%	22.0%	33.0%	27.6%	7.43	2.82	9.21
0.2	0.679159	74,594	97,998	74,515	18.8%	24.5%	33.5%	23.2%	4.30	3.27	10.49
0.28	0.617352	105,782	93,677	69,294	20.6%	27.9%	33.4%	18.1%	3.62	4.08	12.85
0.44	0.532158	145,624	70,266	55,024	24.4%	33.0%	30.9%	11.8%	3.21	6.66	27.08
0.6	0.472464	163,658	44,557	43,953	27.7%	36.1%	27.9%	8.3%	3.22	11.84	800.63
0.606	0.470552	164,103	43,605	43,598	27.8%	36.2%	27.8%	8.2%	3.23	12.14	69,407.20

## Data Availability

The data that support this study are available within the article.

## Appendix A. Proof of Fact 1

We introduce the following proposition, which holds for general coordination number *k* of the Bethe lattice. Particularly, the case k=3 of this proposition is nothing but Fact 1.

**Proposition** **A1.**
*Consider an empty cluster of size greater than 1, and let $m_{j}$ (j=0,1,2,⋯k−1) denote the number of cells of $C_{j}$-type it contains. Then, it holds that*

(A1)
$$\begin{array}{c} m_{k-1}=2+\sum_{\ell =0}^{k-3}\left(k-2-\ell \right)m_{\ell }. \end{array}$$

*Here, the word “a cell of $C_{j}$-type” implies an empty cell with j occupied (and k−j unoccupied) neighbors. Namely, “cells of $C_{0}$-type” and “cells of $C_{1}$-type” are used to denote creating cells and enlarging cells, respectively.*

**Proof.** It is obvious that every empty cluster of size i(i≥2) has at least two $C_{k-1}$-type cells. Then, we choose an arbitrary cell of $C_{k-1}$-type in the empty cluster and call it *P*. And we define *Q* as the unique cell in the cluster adjacent to *P*. It should be noted that *Q* is an empty cell of $C_{j}$-type (0≤j≤k−2) when i≥3 and *Q* is $C_{k-1}$-type only when i=2. If i≥3, then, we replace *P* by an occupied cell and reduce the size of the cluster to i−1. On this reduction, either of the following two changes occurs according to the type of *Q*. If *Q* is $C_{k-2}$-type, then the cluster loses one $C_{k-2}$-type cell and the numbers of cells of other types remain the same. If *Q* is $C_{j}$-type (j=0,⋯,k−3), then the cluster loses one $C_{k-1}$-type cell and one $C_{j}$-type cell. However it gains one $C_{j+1}$-type cell. These observations tell us that every empty cluster of size i(i≥3) is generated from a cluster of size 2. This generation process consists of choosing an occupied cell adjacent to the cluster and replacing it with an empty cell of $C_{k-1}$-type, repeated i−2 times. On each replacement, we have only two possibilities. One is that the number of $C_{k-2}$-type cells increase by one and the numbers of cells of other types remains the same. We call this type of replacement “addition of $C_{k-2}$-type”. The other possibility is that both $C_{k-1}$-type cells and $C_{j}$-type cells (j≤k−3) increase by 1, though $C_{j+1}$-type cells decrease by 1. We call this type of replacement “addition of $C_{j}$-type” (j≤k−3). The empty cluster of size 2 only has two cells of $C_{k-1}$-type and no cells of other types. Hence, for example, if one comes across an empty cluster having four empty cells of $C_{0}$-type, it is realized that the cluster has experienced additions of $C_{0}$-type exactly four times on its generating process. Furthermore, these four additions of $C_{0}$-type consume four cells of $C_{1}$-type, with which the original cluster of size 2 did not equip at all. Hence, this cluster must have experienced additions of $C_{1}$-type at least 4 times. With the same reasoning, this cluster must also have experienced additions of $C_{j}$-type at least four times for each j(j=0,…,k−3). This also implies that the number of cells of $C_{k-1}$-type has increased by at least 4(k−3). Such reasoning applies not only to $C_{0}$-type cells but also to $C_{j}$-type cells (j≤k−3). Hence, as claimed in (A1), any empty cluster containing $m_{j}$ cells of $C_{j}$-type (j=0,⋯,k−3) has exactly $2+\left(k-2\right)m_{0}+\left(k-3\right)m_{1}+\ldots +m_{k-3}$ cells of $C_{k-1}$-type. □

## Appendix B. On the Uniqueness of the Solution of the System of Equations (31)–(36)

We first eliminate the variables $\left\{x_{i},\rho ,n\right\}$ from the set of Equations (31)–(36) and transform the system into a single cubic equation for *T*:

(A2) $$f\left(T\right):=AT^{3}+BT^{2}+CT-D=0,$$

where $A=(11c_{3}+18\delta )$, $B=18\delta^{2}-\delta \left(3c_{2}-7c_{3}\right)-2c_{3}\left(c_{0}+c_{1}\right)-c_{2}c_{3}$, $C=\left(\delta -2c_{1}\right)\left(c_{2}+2\delta \right)\left(c_{3}+3\delta \right)-c_{0}\left(c_{1}c_{3}+2c_{2}c_{3}+6c_{2}\delta +c_{3}\delta \right)$, and $D=c_{0}\left(c_{1}+\delta \right)\left(c_{2}+2\delta \right)\left(c_{3}+3\delta \right)$. This appendix is devoted to showing that this cubic equation has a unique mathematical solution in the open interval (0,1), which is the required domain for *T* according to the definition (19). It is left to Section 5.2 whether this unique solution is physically admissible. In the elimination process, each $x_{i}$ is expressed as

(A3) $$x_{i}=\alpha_{i}x_{0}\;\mathrm{for}\;i=1,2,3,$$

where $\alpha_{1}=3T\left[\left(c_{2}+2\delta \right)\left(c_{3}+3\delta \right)+c_{3}T\right]/M$, $\alpha_{2}=6T^{2}\left(c_{3}+3\delta \right)/M$, $\alpha_{3}=6T^{3}/M$, and $M=\left(c_{1}+\delta \right)\left[\left(c_{2}+2\delta \right)\left(c_{3}+3\delta \right)+c_{3}T\right]+2Tc_{2}\left(c_{3}+3\delta \right)+2c_{3}T^{2}$. We can also recover $\rho ={\left(1+\delta /\langle c\rangle \right)}^{-1}$ with the help of Equation (37), and then recover $x_{0}$ using Equation (31). These explicit expressions assure that the solution $\left\{x_{i},\rho ,n\right\}$ is entirely positive and satisfies 0<ρ<1. Hence, the final hurdle for accepting this unique solution lies in whether $\left\{x_{i}\right\}$ satisfies condition (14). And Section 5.2 investigates this problem.

Now, we proceed to show the existence of the solutions for the cubic Equation (A2). Seeing the expression of *D*, one realizes that f(0)<0. Then, f(1)>0 is also derived under the condition that $0\leq c_{i}\leq 1$ and δ>0. Hence, f(T)=0 must have solutions in (0,1).

To show the uniqueness of the solution, we assume that Equation (A2) has more than one solution in (0,1) and derive a contradiction to condition (14). The assumption implies that *f* takes both the maximum and the minimum in (0,1). Since the derivative of *f* is $f^{\prime }\left(T\right)=3AT^{2}+2BT+C$ and A>0, both B<0 and C>0 are necessary for this assumption. These two inequalities constrain the upper and the lower bound for $c_{0}$; namely, $g_{\mathrm{low}}<c_{0}<g_{\mathrm{up}}$, where

$$\begin{array}{cc} \; & g_{\mathrm{low}}\left(c_{1},c_{2},c_{3}\right):=\frac{-2c_{3}c_{1}-c_{3}c_{2}-3\delta c_{2}+7\delta c_{3}+18\delta^{2}}{2c_{3}},\\ \; & g_{\mathrm{up}}\left(c_{1},c_{2},c_{3}\right):=\frac{\left(\delta -2c_{1}\right)\left(2\delta +c_{2}\right)\left(3\delta +c_{3}\right)}{\delta c_{3}+6\delta c_{2}+2c_{3}c_{2}+c_{3}c_{1}}. \end{array}$$

To make $g_{\mathrm{up}}$ positive, it is required that

(A4) $$\begin{array}{c} \delta >2c_{1}. \end{array}$$

Another requirement is $g_{\mathrm{up}}>g_{\mathrm{low}}$. With the help of inequality (A4), it is noticed that $g_{\mathrm{up}}-g_{\mathrm{low}}$ is a decreasing function with respect to $c_{1}$. Thus, the following must hold:

(A5) $$\begin{array}{cc} \; & 0<g{\left(0,c_{2},c_{3}\right)}_{\mathrm{up}}-g{\left(0,c_{2},c_{3}\right)}_{\mathrm{low}}=:\frac{h(c_{2},c_{3})}{2\left(\delta c_{3}+6\delta c_{2}+2c_{3}c_{2}\right)c_{3}}. \end{array}$$

Here, $h(c_{2},c_{3})$ is explicitly written as

$$\begin{array}{cc} \; & h\left(c_{2},c_{3}\right)=2{\left(3\delta +c_{3}\right)}^{2}{c_{2}}^{2}-\delta \left(11c_{3}+36\delta \right)\left(3\delta +c_{3}\right)c_{2}-3c_{3}\delta^{2}\left(c_{3}+2\delta \right), \end{array}$$

which is a quadratic polynomial in $c_{2}$. Inequality (A5) requires $h(c_{2},c_{3})$ to be positive. For this purpose, $c_{2}>5\delta$ must be satisfied, because an explicit calculation shows that $0>h\left(5\delta ,c_{3}\right)>h\left(4\delta ,c_{3}\right)$. Meanwhile, $g_{\mathrm{up}}$ is a decreasing function with respect to $c_{2}$ and one can directly check that $c_{0}<g_{\mathrm{up}}\left(c_{1},5\delta ,c_{3}\right)<5\delta$ holds. Hence, $c_{0}<c_{2}$. This implies $x_{0}>x_{2}$ when considered with Equation (33), which contradicts the condition (14).

## Appendix C. Explicit Formulas for *X_i_*, *I* = 0,1,2,3

$$\begin{array}{ccc} x_{0} & = & \frac{1}{\Delta }[\left(3c_{1}c_{2}-15c_{1}c_{3}+12c_{2}c_{3}-12c_{1}\delta +16c_{2}\delta -4c_{3}\delta \right)\delta n+\left(-9c_{1}c_{2}c_{3}-18c_{1}c_{2}\delta +12c_{1}c_{3}\delta -2c_{2}c_{3}\delta \right)N+\\ & & +(9c_{1}c_{2}c_{3}+22c_{1}c_{2}\delta -20c_{1}c_{3}\delta +15c_{2}c_{3}\delta +16c_{2}\delta^{2}-8c_{3}\delta^{2})N\rho ],\\ x_{1} & = & \frac{1}{\Delta }[\left(-5c_{0}c_{2}+17c_{0}c_{3}-12c_{2}c_{3}+12c_{0}\delta -18c_{2}\delta +6c_{3}\delta \right)\delta n+\left(19c_{0}c_{2}c_{3}+36c_{0}c_{2}\delta -18c_{0}c_{3}\delta \right)N+\\ & & +(-19c_{0}c_{2}c_{3}-43c_{0}c_{2}\delta +19c_{0}c_{3}\delta -13c_{2}c_{3}\delta -12c_{0}\delta^{2}-18c_{2}\delta^{2}+12c_{3}\delta^{2})N\rho ],\\ x_{2} & = & \frac{1}{\Delta }[\left(2c_{0}c_{1}-17c_{0}c_{3}+15c_{1}c_{3}-16c_{0}\delta +18c_{1}\delta -2c_{3}\delta \right)\delta n+\left(-11c_{0}c_{1}c_{3}-18c_{0}c_{1}\delta +6c_{0}c_{3}\delta \right)N+\\ & & +(11c_{0}c_{1}c_{3}+22c_{0}c_{1}\delta -7c_{0}c_{3}\delta +8c_{1}c_{3}\delta +16c_{0}\delta^{2}-4c_{3}\delta^{2})N\rho ],\\ x_{3} & = & \frac{1}{\Delta }[\left(-2c_{0}c_{1}+5c_{0}c_{2}-3c_{1}c_{2}+4c_{0}\delta -6c_{1}\delta +2c_{2}\delta \right)\delta n+\left(c_{0}c_{1}c_{2}+6c_{0}c_{1}\delta -4c_{0}c_{2}\delta \right)N+\\ & & +(-c_{0}c_{1}c_{2}-10c_{0}c_{1}\delta +11c_{0}c_{2}\delta -4c_{1}c_{2}\delta -4c_{0}\delta^{2}+2c_{2}\delta^{2})N\rho ],\\ \Delta & = & c_{0}c_{1}c_{2}-11c_{0}c_{1}c_{3}+19c_{0}c_{2}c_{3}-9c_{1}c_{2}c_{3}+\delta \left(-12c_{0}c_{1}+32c_{0}c_{2}-18c_{1}c_{2}-12c_{0}c_{3}+12c_{1}c_{3}-2c_{2}c_{3}\right). \end{array}$$
